# Effects of mobile learning on writing panoramic radiograph reports: a quasi-experimental trial in dental education

**DOI:** 10.1186/s12909-021-02889-0

**Published:** 2021-09-01

**Authors:** Anna Bock, Dirk Elvers, Florian Peters, Chris Kramer, Kristian Kniha, Frank Hölzle, Cord Spreckelsen, Ali Modabber

**Affiliations:** 1 Department of Oral and Maxillofacial Surgery, University Hospital of Aachen University, Pauwelsstr. 30, 52074 Aachen, Germany; 2 Department of Medical Informatics, RWTH Aachen University, Pauwelsstr. 30, 52074 Aachen, Germany; 3 Institute of Medical Statistics, Computer and Data Sciences, University Hospital Jena, Nachstraße 18, 07743 Jena, Germany

**Keywords:** Dental education, Dental radiology, Mobile application, M-learning, Mobile technology

## Abstract

**Background:**

In dentistry, the reporting of panoramic radiographs is particularly challenging, as many structures are depicted in one image and pathologies need to be identified completely. To enhance the learning process for these interpretations, the advantages of the increasingly popular education method of mobile learning could be used. Therefore, this study aimed to determine the effectiveness of learning to report panoramic radiographs using an application (app) on a mobile device.

**Methods:**

The existing e-learning programme ‘PantoDict’ was further developed into a mobile app with a new training section. Participants of a dental radiology course were divided into two groups, one of which additionally had the chance to practise reporting panoramic radiographs using the app. A test to assess the knowledge gained was conducted at the end of the semester; the course and the app were also evaluated.

**Results:**

The group that used the app showed significantly better results in the test than the control group (*p* < 0.05). Although the app group approved a high satisfaction using the app as an additional supplement to the course, this did not result in a higher overall satisfaction with the course. Further, these students observed that the traditional face-to-face seminar could not be replaced by the app.

**Conclusion:**

By using the PantoDict app, students were offered better training options for writing reports on panoramic radiographs, which resulted in significantly better test results than the results of the control group. Therefore, the mobile app is a useful supplement to classical education formats within the context of a blended learning approach.

**Supplementary Information:**

The online version contains supplementary material available at 10.1186/s12909-021-02889-0.

## Background

Mobile technology and its advantages have become a pivotal aspect of the modern world. In recent years, the use of mobile phone applications (apps) has dramatically increased, even among health care professionals. The use of mobile apps in clinical practice has several advantages, for example, the facilitation of communication, portability and efficient time use [1, 2]. Further, it has been suggested that the implementation of mobile devices and associated app use has led to increased productivity and access to clinical information at the point of care [3, 4]. Consequently, scientists have begun investigating the role of mobile technology in both work and education [5].

Mobile learning (m-learning) generally describes all forms of learning with mobile devices, such as smartphones or tablets. Currently, many students own at least one mobile device, are able to use it and perceive it as a learning tool [6, 7]. Compared to traditional computers, mobile devices present educational content in a similar manner. However, a major advantage of m-learning is ubiquitous access [8]. Such access provides students a self-directed learning environment, which is independent from temporal and physical limitations. Furthermore, mobile apps can be used offline, which generates constant access to educational content and knowledge [6, 9]. Consequently, m-learning enables a form of learning that is more autonomous and individual than classical teaching formats [9]. In turn, this can enhance theoretical knowledge, clinical competency and confidence among medical students [10]. In general, m-learning is increasingly popular among and appreciated by medical students [11–13].

M-learning can be particularly useful to access digital educational content in the context of blended learning concepts-a combination of digital media and traditional classroom teaching [14]. Using the mobile devices, the digital learning content can be assessed easily for preparation of the class, as flipped classroom model or for follow-up of the learning materials [15].

Radiographic examinations are an essential part for diagnosis and treatment planning in dentists’ daily clinical practice. Since the interpretations of radiographic images are usually done by the dentists themselves, the skills needed to complete image reporting are taught during dental education. Panoramic radiographs are one of the imaging techniques most often used in clinical practices. The reporting of these radiographs are particularly challenging, as many structures are depicted in one image and pathologies need to be identified completely. To improve and facilitate the learning process on how to report panoramic radiographs, a mobile app to learn and train this process has been developed. As part of a blended learning concept for the dental radiology class, the app has been added as supplementary digital learning tool to the traditional face-to-face class at our university.

This study aimed to determine the effectiveness of learning on writing panoramic radiograph reports using a mobile app. Therefore, the knowledge gain of a group of students that attended a typical seminar was compared to a group that attended the seminar and additionally used an app to practise. Moreover, the study also aimed to assess the students’ satisfaction with using the app.

## Methods

### PantoDict app

The German ‘PantoDict’ progressive web app is based on the e-learning programme ‘PantoDict’, which was developed 2018 by the department for medical informatics RWTH Aachen University [16]. PantoDict has a tutorial and a training section; the tutorial section includes step-by-step instructions for the user interface. A video demonstration and two written reports, prepared for reporting panoramic radiographs, were utilised as training tools [16]. The training section of the e-learning programme has been further developed; thus, the section now consists of the following two parts. For both parts, only images with good quality and easily identified anatomic landmarks and abnormalities were chosen.

**Fig. 1:**
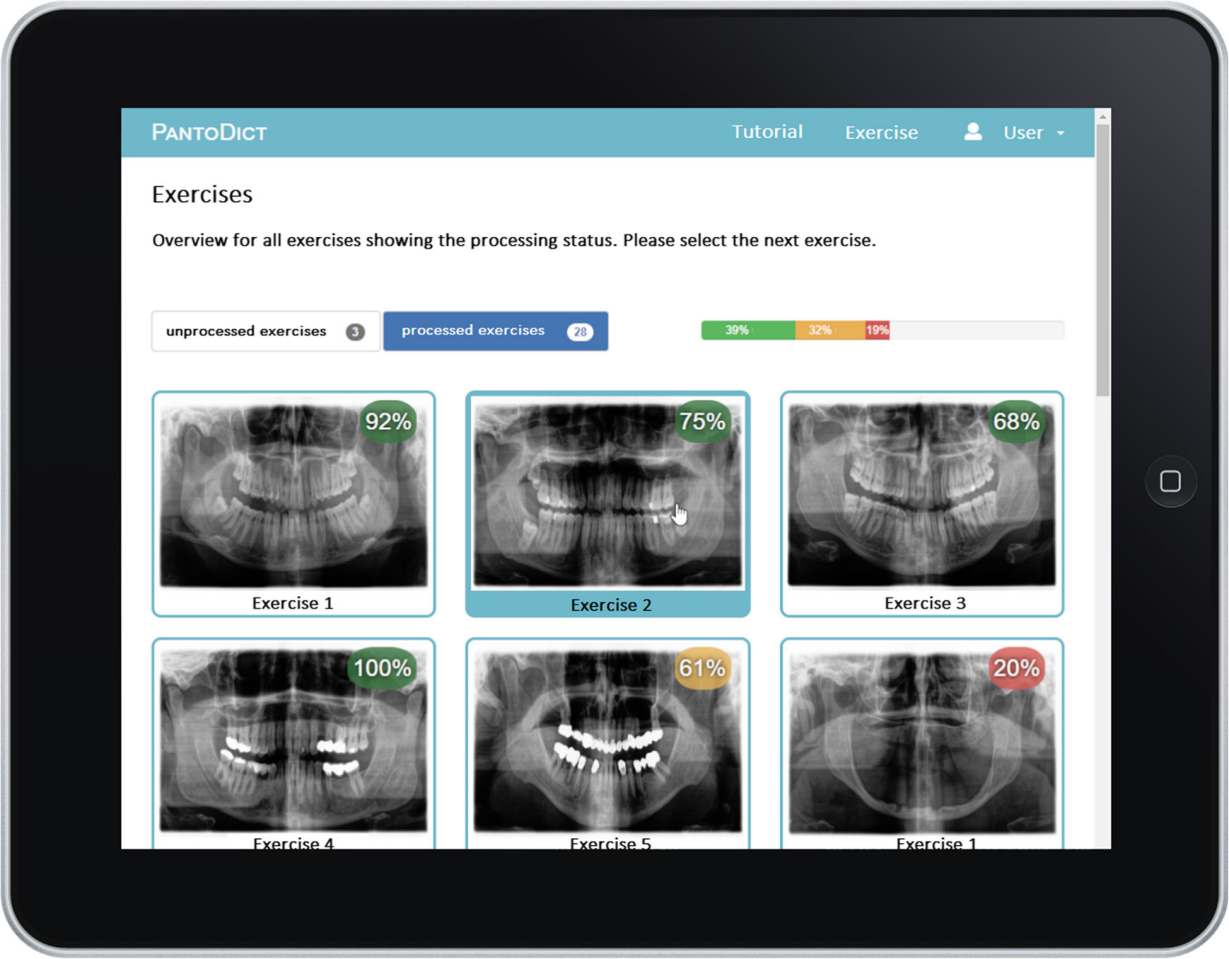
Overview of the training section displaying the processing status of each reporting exercise in percentage, indicating the overall score of the student’s interpretation in the upper right margin

**Fig. 2:**
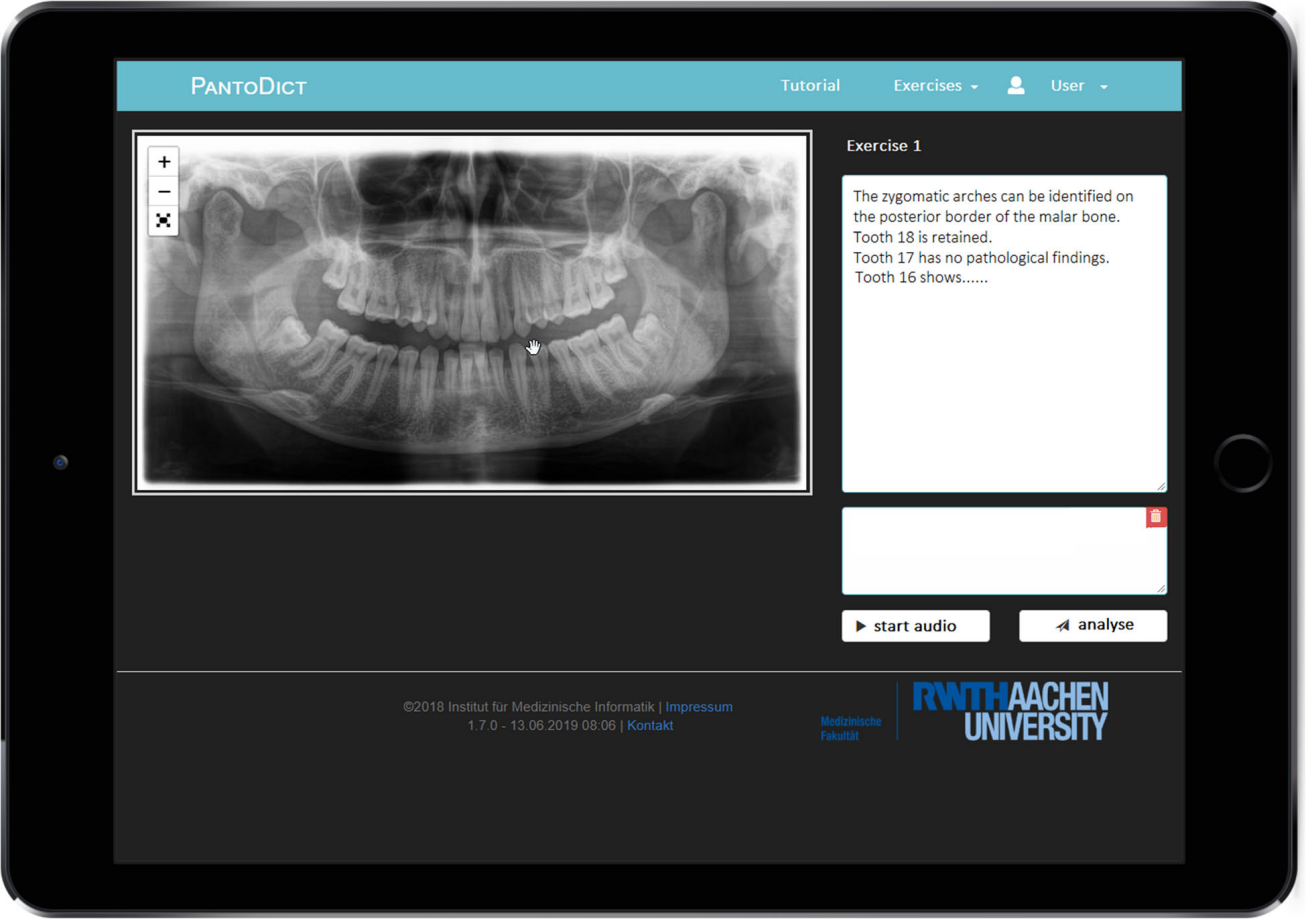
Screen view showing a reporting exercise in process. The radiograph can be zoomed in and out to observe details. The right upper text field contains the entered report. The second text field shows the recognized text while using automatic speech recognition and can also be used to correct the text. Once the report is completed, the analysation can by started by pressing the button‘analyse’

One part contains the report exercises wherein 30 radiographs with different pathologies were provided to generate reports, as shown in Fig. 1. For example, the images present fractures, osteosynthesis, cysts, osteomyelitis, and artificial teeth. The reports of the selected radiograph cases were entered using automatic speech recognition. Subsequently, the text could be edited, and spelling errors could be corrected using the keyboard (Fig. 2). Once a report was completed, the text was analysed and evaluated automatically. In the performance summary, students could compare their own reports with an ideal report of the case. The score of the report was displayed as a percentage [16].

**Fig. 3:**
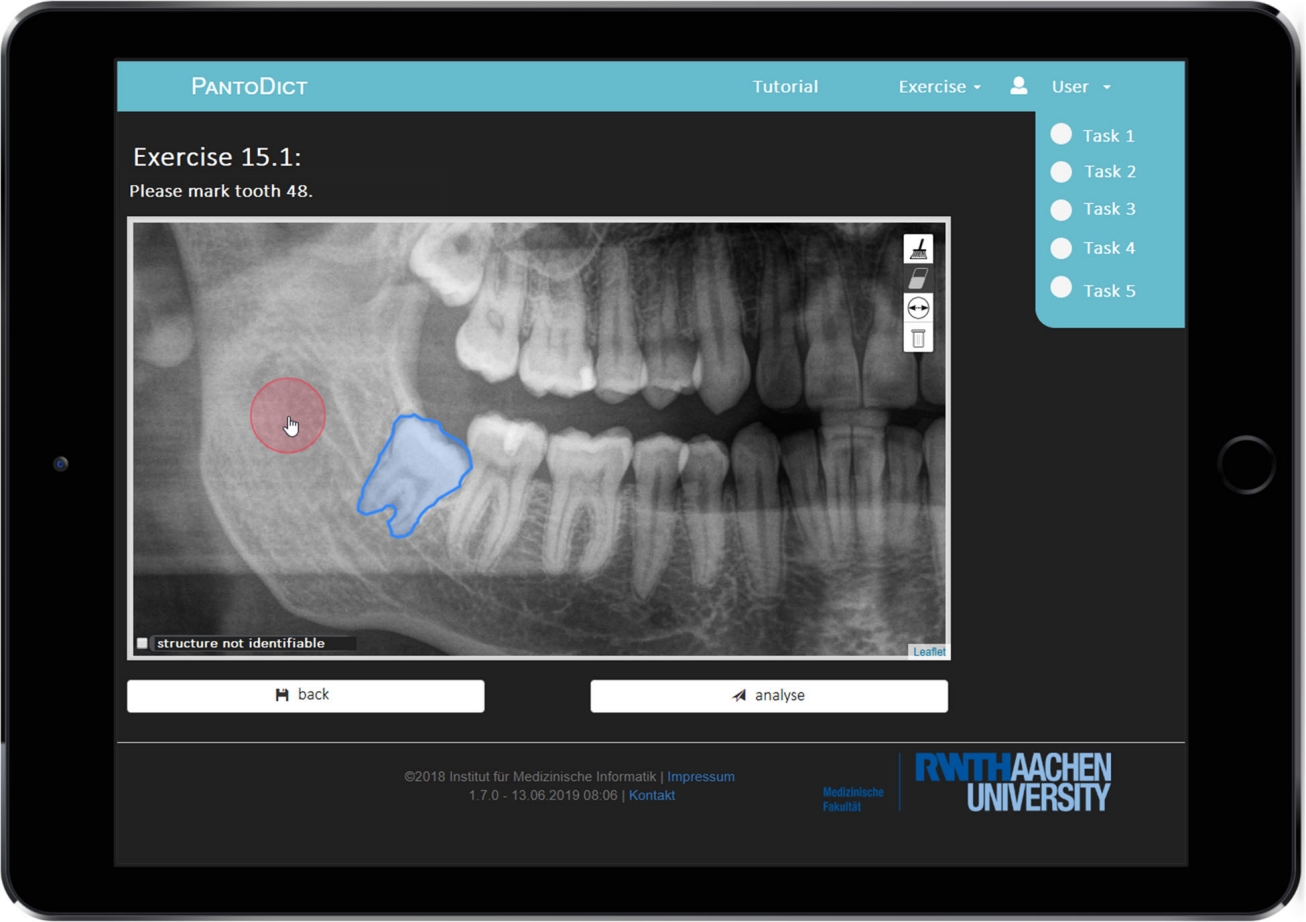
Screen view showing a marking exercise in process. There are always 5 tasks for each radiograph. In this example, the participant is asked to mark the tooth 48

The new part of the training section, developed for the app, contains marking exercises wherein another 30 panoramic radiographs of teeth with different pathologies were integrated. There were five tasks for each panoramic radiograph. In each task, the user was requested to mark an anatomical landmark or a pathological structure in the radiograph, as shown in Fig. 3. All marking exercises were validated by the course leaders. In the performance summary, students could compare their drawing in the radiograph to the ideal marking of the case. All results were displayed as percentages.

Technically, PantoDict is a web app, that is, an application provided by a web server and delivered by a web browser installed on the (mobile) device of the learner. The server module was a Java Spring application [17]. It used the Java Persistence API [18] and the MySQL [19] database management system to enable data persistence. To ensure the flexibility of the database schema during the development process and for future versions, the Liquibase library [20] was adopted. The client module was based on JavaScript. It includes libraries, which were originally developed for processing the geospatial data (Turf) [21] to support the interactive (graphical) specification of regions of interest in the panoramic radiographs and the algorithmic comparison with the respective gold standard provided by the experts. PantoDict used HTML5 Speech Recognition API for transcribing spoken reports [22].

To provide a good view on the panoramic X-ray on a mobile device, there is a zoom function with a high resolution. The scoring of the first training section, the panoramic radiograph reports, was based on the following three steps. First, the order of the statements concerning individual teeth was standardised using regular expressions. The second step involved performing terminological standardisation (including synonym detection) using the Metathesaurus of the Unified Medical Language System [22, 23]. Finally, the third step scored the similarity based on part-of-speech tagging and term comparison. Text processing adopted a UIMAfit-Pipeline including DKpro-Modules [24, 25]. The scoring of the marking exercises were achieved by comparing the percentual overlapping of the markings.

### Participants

All students of the dental radiology course were invited to take part in the study (*n* = 38). The participation was voluntary and the score of the test did not influence their mark for the course. Informed consent was obtained from each participant. All methods were carried out in accordance with relevant guidelines and regulations.

### Study design

The study took place during the summer academic term of 2019 from April to July. All participants of the course (*n* = 38) attended two seminars to learn how to make reports on panoramic X-rays. The two seminars took place 4–6 weeks apart. The duration of all seminars was 90 min. Each seminar comprised a group of six students. The creation of a panoramic X-ray report was demonstrated by the lecturer at the beginning of the first seminar for each group. The lecturers of the seminars were independent from the research to prevent a bias. Subsequently, each student had to interpret a panoramic radiograph report out loud to the other participants, describing all the structures and pathologies. Feedback on the report was given immediately by the lecturer. Accordingly, six panoramic radiograph reports were discussed in each seminar. In this model, each participant learned how to compose panoramic radiograph reports by reporting one on their own and by observing the reports of other participants. The interaction within the seminar groups was intended as part of the learning process.

**Fig. 4:**
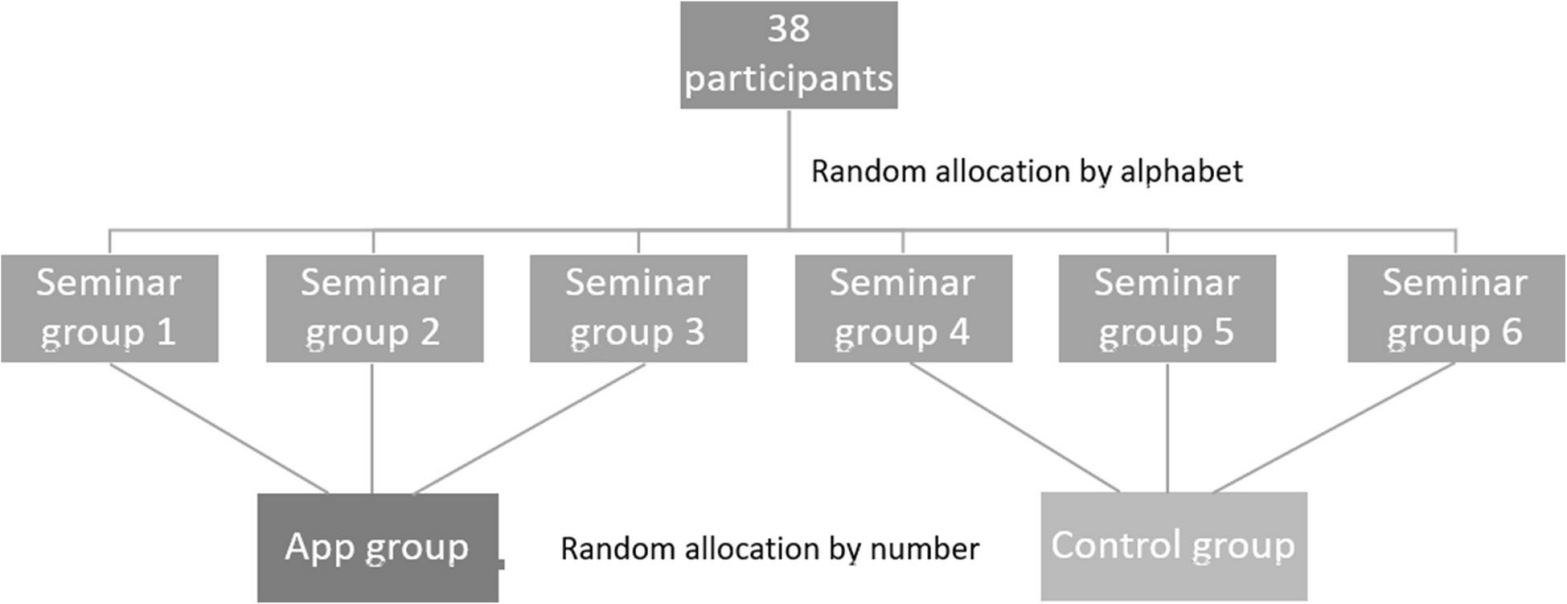
Allocation of the participants into the seminar groups and subsequently into the app and control group

All students were assigned to the six groups one after the other according to the alphabetical order of their last names.. Subsequently, the seminar groups were allocated to the app or the control group in numerical order (see Fig. 4). The app group (*n* = 19) got login details and had access to the PantoDict app from the beginning of the semester. These students were asked to further practice creating reports on panoramic radiographs through the app within a blended learning concept. The amount of autonomously practise was up to themselves. The usage of the app was tracked anonymously. The other group (*n* = 19) served as a control group and only practised reporting panoramic radiograph in the seminar. The displayed panoramic radiographs of the seminar were different than the ones used in the app, although the pathologies were similar. By keeping the seminar groups through the term, an in class interaction of the two groups with each other was supposed to be prevented.

### Data collection and measurement tools

At the end of the semester in July, a test was conducted to evaluate whether app use had an influence on learning achievement. In the test, students had to write a complete report on a panoramic radiograph. A proofing sheet, which was validated by the two course leaders, was used to evaluate the report. The validation was carried out in such a way, that both course leaders performed the report themselves and then the reports were merged in the proofing sheet. The proofing sheet consists of two parts. The first part lists all potentially visible structures of a panoramic X-ray, including structures like soft tissues (i.e. the shade of the soft palate) or bony structures (i.e. mandibular joint). In order to examine a panoramic radiograph completely, all these structures have to be evaluated in the report. The second part regards general aspects like using the correct terminology and order of the report. Each correct statement was assessed with one point; there were no half points or negative points. The maximum test score was 100 points. All participants had to evaluate their satisfaction with the course. Besides that, they had to evaluate their subjective self-assessment on knowledge and confidence on reporting panoramic radiographs; students who used the app also had to evaluate PantoDict itself. Besides that, students using the app had to indicate which of the two types of exercises they prefer. Finally, all students were asked to respond to 2 open-ended questions asking what they liked about the seminars and what could be improved (see Supplementary Material). The evaluation questions were answered based on a 10-point Likert scale, with 1 indicating ‘fully agree/very satisfied’ and 10 indicating ‘totally disagree/unsatisfied’.

### Statistics

The obtained data were arranged using MS Office Excel 2016® (Microsoft Corporation, Redmond, Washington, USA). Statistical analyses were conducted using GraphPad Prism 6 Software (GraphPad Software, San Diego, CA). An unpaired t-test was used to compare the test results of both groups after normal distribution was checked through a D’Agostino–Pearson normality test in omnibus K2 variant. *P* ≤ 0.05 was considered statistically significant. The effect size for discriminating between groups was estimated using Cohen’s *d* effect size and represented as *d* in the Results section. Values were defined as small (0.20–0.49), medium (0.50–0.79), large (0.80–1.29), and very large (above 1.30) [16].

## Results

### Study participants

Of all participants (*n* = 38), 18 were women and 20 were men. Thirteen participants were between 19 and 21 years old, 15 were between 22 and 24 years old and 10 were older than 24 years.

### Usage data app

**Fig. 5:**
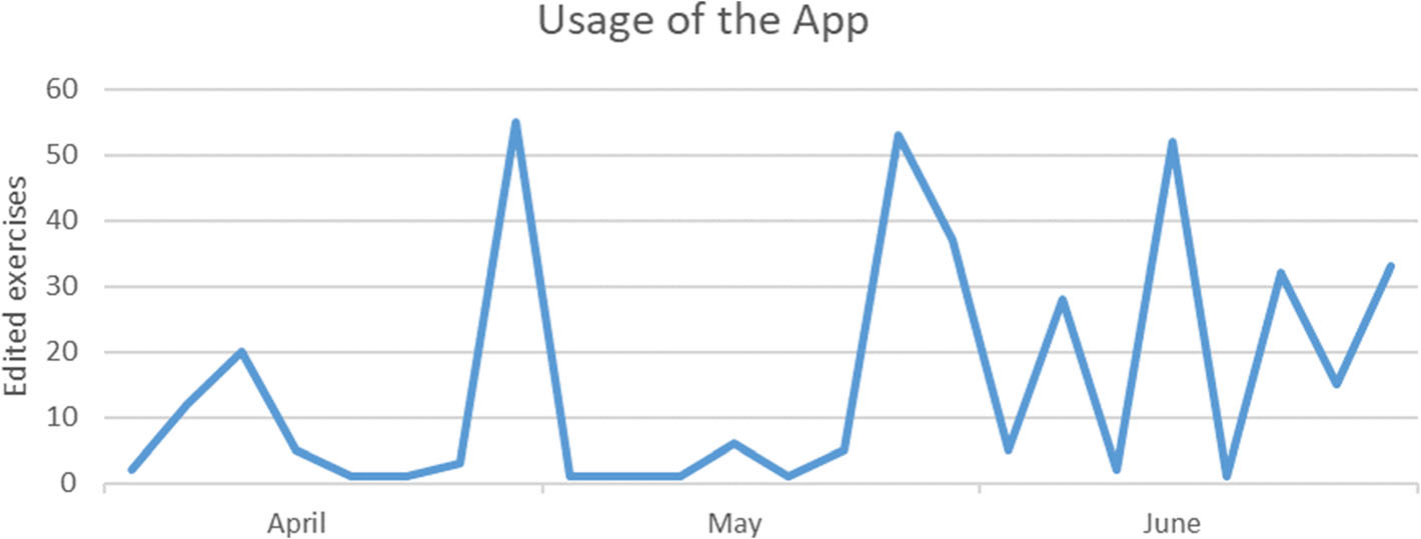
Monthly app usage for the summer semester

During the entire semester, 394 exercises (377 marking exercises and 17 report exercises) were edited by the group using the app. The mean number of edited exercises for each student (*n* = 19) was 24.6 (SD = 21.33). Use of the app increased prior to the end of the semester and examinations, as illustrated in Fig. 5.

### Test

**Fig. 6:**
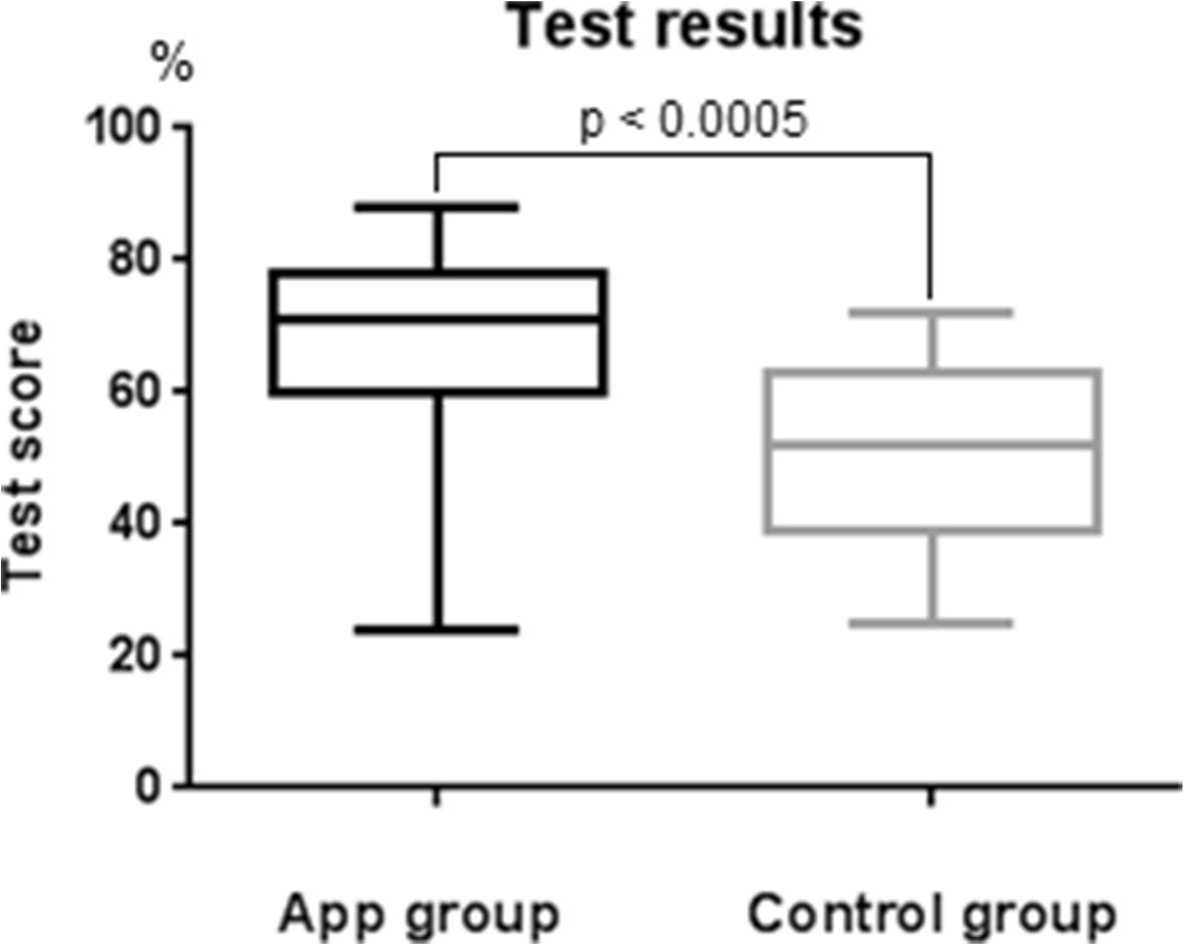
Comparison of the results of the group that used the PantoDict app and the control group. The app group showed significantly better results in the test than the control group

The mean test scores were 68.63% (SD = 15.16) and 50.21% (SD = 13.83) for the group that used the PantoDict app and the control group, respectively. There was a significant difference in the test scores for both groups (*p* < 0.0001). All results are displayed in Fig. 6.

### Evaluation

Regarding the self-assessment, all students of both groups confirmed that they gained knowledge for writing a report on a panoramic radiograph through the course. The mean score of the self-assessment for the group that used the app was 2.21 (SD = 1.06), and the mean score for the control group was 2.32 (SD = 1.72). The difference of the self-assessment score between the groups was not significant (*p* >  0.05; *d* = 0.077). After attending the course, the students who used the app to practise gained more confidence in reporting panoramic radiographs (mean score = 3.42; SD = 1.04) than the control group (mean score = 4.1; SD = 1.86). The difference was not significant (*p* >  0.05; d = 0.451). Overall, students of both groups were satisfied with the course. The mean score of satisfaction for the group that used the app was 2.53 (SD = 1.6), and the mean score for the control group was 2.37 (SD = 1.18). The difference was not significant (*p* >  0.05; d = − 0.114). All results are shown in Table 1.

**Table 1:** Results of the students evaluation of both groups regarding self-assessment, confidence gain and satisfaction with the entire course. All aspects were evaluated on a ten-point Likert scale, with 1 indicating ‘fully agree/very satisfied’ and 10 ‘totally disagree/unsatisfied’

Evaluated Aspect	App group mean score	Controll group mean score	Statistical difference (*p*)
Self-assessment	2.21 (SD = 1.06)	2.32 (SD = 1.72)	> 0.05
Gain of confidence	3.42; SD = 1.04	4.1; SD = 1.86	0.18
Satisfaction with the course	2.53 (SD = 1.6	2.37 (SD = 1.18)	> 0.05

Participants of the group that used the app expressed a high satisfaction with the additional training options and the blended learning concept. The students agreed that they could imagine learning through the app on a regular basis in the future (mean score = 1.84; SD = 1.09) and that they would like to use the app on a constant basis (mean score = 1.37; SD = 0.98).

The students using the app confirmed that they gained knowledge in relation to writing panoramic radiograph reports through the app (mean score = 2.53; SD = 1.35) and that the app was substantially involved in their consolidation skills (mean score = 1.84; SD = 1.14). Nevertheless, they did not agree that the app was superior to the seminars (mean score = 5.26; SD = 2.15) concerning the knowledge transfer on reporting panoramic radiographs Instead, the pointed out, that the PantoDict App is a good supplement to the seminars (mean score = 1.21; SD = 0.52). With respect to the two different types of exercises, an almost equal number of students preferred the reporting (*n* = 9) and marking exercises (*n* = 10).

All results of the evaluation are shown in Table 2.

**Table 2:** Results of the app evaluation. All aspects were evaluated on a ten-point Likert scale, with 1 indicating ‘fully agree/very satisfied’ and 10 ‘totally disagree/unsatisfied’

Evaluated aspect	Mean score	Standard deviation
The usage of the PantoDict App helped to improve my knowledge on reporting panoramic radiographs significantly.	2.53	1.35
The PantoDict App was substantially involved in the consolidation of my reporting skills.	1.84	1.14
Concerning the knowledge transfer, the PantoDict App is superior to the seminars.	5.26	2.15
The PantoDict App is a good supplement to the seminars.	1.21	0.52
The PantoDict App is well structured.	2.1	1.07
The learning contents were easy to understand.	1.84	0.99
The PantoDict App is didactically well designed.	1.84	0.99
The PantoDict App has an intuitive interface.	1.89	1.29
PantoDict App motivates me to learn more about reporting panoramic radiographs.	3.26	2.57
I would like to use the PantoDict App in the future.	1.84	1.08
I would like to use PantoDict App all the time.	1.36	0.98

In the open-ended questions, students of the conventional group requested more seminars to learn how the report panoramic radiographs better. Students of the group using the app, liked the interactivity and the immediate feedback of the programme. Besides that, they appreciated the quantity of images and the easy handling of the app. One person claimed that the processing of synonyms could be improved.

## Discussion

The e-learning programme PantoDict serves as a tool for students to practise analysing and reporting panoramic radiographs through two different types of exercises. It has been further developed as an app for mobile devices and established as a supplementary learning tool to traditional face-to-face teaching in dental radiology at our university. In this way, the blended learning concept enables students to take individual learning style and speed into account and to study independently from the constraints of time and place [26, 27]. Besides that the advantages of m-learning can be used. In this study, the flipped classroom approach is also used, as students have the opportunity to practice reportings before the seminars and then clarify ambiguities in the seminars.

Our study showed that the blended learning approach resulted in significantly better test scores than the control group. This confirms the results of other studies, which have shown that independent digital learning is very efficient [26, 28]. Nonetheless, students of the blended group were offered more options to practise reporting panoramic radiographs. This resulted in a higher number of examined radiographs by each participant in the group using the mobile application compared to the students that examined radiographs only in the seminars. With respect to the usage data, each participant using the app reported about one more panoramic radiograph than the other group. Additionally, the app group had the opportunity to deepen their knowledge on analysing panoramic radiographs with the marking exercises, which queried the gained skills in a different way. Approximately 20 marking exercises were processed by each participant. These bias of unequal practising conditions could influence the results of the final test to an important extent.

Regarding the self-assessment of knowledge gain on reporting panoramic radiographs, both groups assessed their skills equally. Nevertheless, in this study the blended learning approach resulted in a better learning outcome compared to the control group. Other studies have shown similar results with better test scores [29, 30]. In this study, regardless of whether the app was used or not, both groups were equally satisfied with the course. Although the app group approved a high satisfaction using the app as an additional supplement to the course, this did not result in a higher overall satisfaction with the course. This finding is contrary to findings of other studies, where the blended learning concept resulted in a higher student satisfaction [29, 30].

Supportive technology offers great value for the learning process, as individual needs can be better taken into account and additional training options can be offered in a crowded curriculum. With the further development of the e-learning programme as a mobile device application, it offers additional advantages. One major advantage is the ubiquitous access of the app. Students can practice at any time and in any place, for example, on their way home in the bus or train. The app can be used easily, even within a limited time period. Another advantage is that the app can be used offline. Therefore, it is independent from internet or mobile phone reception [9]. In addition, the application offers a very high operating comfort. It enables an easier use of system functions such as the microphone in comparison to regular computers, which usually need additional equipment such as a headset for automatic speech recognition. Moreover, mobile applications can be used as an educational tool with an equal effectiveness compared to that of other teaching methods, such as patient simulators [31].

Overall, students’ evaluations of the app were considerably positive. They expressed a very high satisfaction with respect to all aspects of the programme, particularly in terms of the structure of the app, didactical concept and intuitive processing. Moreover, the participants indicated that they would like to use the app at any given time.

However, there are also some disadvantages of m-learning. First, it is completely reliant on technology. There is always the possibility that technology may fail or may be inadequate. Owing to continuing technological developments, these issues play a subordinate role at present. Furthermore, there is always the risk of distraction when studying with a mobile device, as mobile phones are used for numerous other functions such as calls or messages and may interrupt a study session. Moreover, ubiquitous access to study and practice may cause some problems. There could be an issue of misuse or overuse due to its accessibility and availability [9].

Another important aspect is the high amount of work required to generate a blended learning approach. The entire process to develop the mobile app consumes a substantial amount of time, manpower and expenses. Nonetheless, a study by Maloney et al. demonstrated that a blended learning approach was more cost-effective to operate. After the third year iteration, it resulted in improved value for the concerned institution [32].

The evaluation showed that according to students’ opinions, the app was not superior to regular face-to-face seminars. The students observed that it was more akin to a useful supplement to the course. This underlines the findings of other studies in that personal interaction cannot be replaced by e-learning. Students participating in online courses mentioned that they missed personal interaction; in addition, they indicated isolated feelings and a weak sense of community [33, 34].

The app’s usage analysis underlines the finding of the app being a useful supplement to the course in a blended learning concept. At the beginning of the semester, there were relatively few logins and edited exercises because the students had no prior knowledge on how to write a report for a panoramic radiograph. After the first seminar, the usage rate increased immediately. The participants intended to apply their newly gained knowledge and to practise reporting. The highest usage observed was towards the end of the semester, which is likely due to the imminent examination.

The study has some limitations. One limitation is that the panoramic radiographs used in the app were different from the panoramic radiographs used in the seminars. By using images in the seminars that are updated daily, it is the most likely way of providing students with realistic working conditions. The app however, provides a various set of images including the most important pathologies. Like this, students can be offered more training options and a complete overview of pathologies on panoramic radiographs. As mentioned before, this additional training offer can lead to a bias of the test results between the groups. Another limitation is that the interaction of the two groups during the semester cannot be controlled. It is possible for the students to exchange information about the course content and also access data for the app, which could result in falsified results.

## Conclusion

By using the PantoDict app, students were offered better training options for reporting panoramic X-rays, which resulted in significantly better test results than those of the control group. Although the app group approved a high satisfaction using the app as an additional supplement to the course, this did not result in a higher overall satisfaction with the course. Further, these students observed that the traditional face-to-face seminar could not be replaced by the app. Therefore, the app could provide a potentially useful supplement to classical education formats within the context of a blended learning approach. In future research the long-term outcome of using the mobile app as well as the actual utilisation rate through the year could be evaluated.

## Supplementary Information

**Additional file 1.** . Proofing sheet.

## Data Availability

The datasets used and/or analysed during the current study are available from the corresponding author on reasonable request.
