# Supplementary material for: Effects of mobile learning on writing panoramic radiograph reports: a quasi-experimental trial in dental education
Source: BMC Med Educ. 2021 Sep 1;21:466. doi: 10.1186/s12909-021-02889-0 (PMC8411548; doi:10.1186/s12909-021-02889-0)
Supplement: Supplementary file 1 — Additional file 1. . Proofing sheet. [file 12909_2021_2889_MOESM1_ESM.docx]

**Proofing sheet**

|  |  | Points | Assessment |
| --- | --- | --- | --- |
| 1 | It is a panoramic radiograph with correct exposure. | 1 |  |
| 2 | The patient is positioned too far posteriorly so that the chin and left temporomandibular joint are partially cut off. | 1 |  |
| 3 | The articular tuberosity is identifiable on both sides and flattened anteriorly. | 1 |  |
| 4 | The condyles are centric in both articular fossae. | 1 |  |
| 5 | When compared from the side, the right joint space is slightly more narrow than on the left side. | 1 |  |
| 6 | The left maxillary sinus is more shaded than the right sinus. | 1 |  |
| 7 | In the right sinus there is a semicircular, basal and medial, homogeneous shadow that extends from region 17-14. | 1 |  |
| 8 | The lower jaw shows an intact boundary with no signs of a continuity interruption. | 1 |  |
| 9 | The hyoid bone is cut off on both sides at the lower edge of the picture. | 1 |  |
| 10 | The tongue was not placed on the palate during recording, so that there is a tongue shadow. | 1 |  |
| 11 | The soft palate is good on the left and partially visible on the right. | 1 |  |
| 12 | The anterior border of the pharyngeal shadow is shown on both sides. | 1 |  |
| 13 | The caudal portion oft he right ear can be identified. | 1 |  |
| 14 | The nasal septum and the inferior naal conchae are visible in the main nasal cavity. | 1 |  |
| 15 | The zygomatic arches can be identified on the posterior edge of the zygomatic bodies. | 1 |  |
| 16 | The mandibular formaina are visible on both sides. | 1 |  |
| 17 | The canalis mandibulae can be identified on both sides in ist course up to the 2^nd^ molar. | 1 |  |
| 18 | The mental foramen on the left can be identified mesial to the root of tooth 35. The mental formaen on the right can be identified caudally to the tip of the root of tooth 44. | 2 |  |
| 19 | No bone degradation in the alveolar process was observed in either the upper or lower jaw. | 1 |  |
| 20 | Tooth 18 is retained mesially, the tip of the root cannot be assessed. | 2 |  |
| 21 | Tooth 17 has a whitening mesially, an addition effect mesially and the root tip cannot be assessed | 3 |  |
| 22 | Tooth 16 has an occlusal metal-tight shadowing, distal and mesial an addition effect and the root tip cannot be assessed | 3 |  |
| 23 | Tooth 15 has distal and mesial an addition effect; the root tip cannot be assessed | 2 |  |
| 24 | Tooth 14 has distal and mesial an addition effect; the root tip cannot be assessed | 2 |  |
| 25 | Tooth 13 has distal and mesial an addition effect; the root tip cannot be assessed | 2 |  |
| 26 | Tooth 12 has distal an addition effect, the root tip is without pathological findings | 2 |  |
| 27 | Tooth 11 has incisal an addition effect due to bite on exposure, the root tip cannot be assessed | 2 |  |
| 28 | Tooth 21 has incisal an addition effect due to bite on exposure, the root tip cannot be assessed | 2 |  |
| 29 | Tooth 22 the root tip is without pathological findings | 1 |  |
| 30 | Tooth 23 has distal an addition effect, the root tip is without pathological findings | 2 |  |
| 31 | Tooth 24 has distal and mesial an addition effect; the root tip cannot be assessed | 2 |  |
| 32 | Tooth 25 has distal and mesial an addition effect; the root tip cannot be assessed | 2 |  |
| 33 | Tooth 26 has a small whitening distal, an addition effect mesial and distal, the root tip cannot be assessed | 3 |  |
| 34 | Tooth 27 has mesial an addition effect; the root tip cannot be assessed | 2 |  |
| 35 | Tooth 28 the root tip cannot be assessed | 1 |  |
| 36 | Tooth 38 is retained and horizontally displaced mesially, has a small crescent-shaped lightening between the tooth crown and the mesio-caudal bone, the tip of the root cannot be assessed. | 3 |  |
| 37 | Tooth 37 has a metal-tight occlusal shading, a lightening distal, the root tip cannot be assessed | 3 |  |
| 38 | Tooth 36 has a brightening mesial, an addition effect mesial, the root tip cannot be assessed | 3 |  |
| 39 | Tooth 35 has a whitening distal, distal and mesial an addition effect, the root tip cannot be assessed | 3 |  |
| 40 | Tooth 34 has distal an addition effect, the root tip cannot be assessed | 2 |  |
| 41 | Tooth 33 the root tip cannot be assessed | 1 |  |
| 42 | Tooth 32 the root tip cannot be assessed | 1 |  |
| 43 | Tooth 31 has incisal an addition effect due to bite upon exposure, an addition effect mesially, the root tip cannot be assessed | 3 |  |
| 44 | Tooth 41 has incisal an addition effect due to bite upon exposure, an addition effect mesially and distally, the root tip cannot be assessed | 3 |  |
| 45 | Tooth 42 incisal addition effect due to bite upon exposure, addition effect mesially and distally, the root tip cannot be assessed | 3 |  |
| 46 | Tooth 43 has an addition effect mesially and distally, the root tip cannot be assessed | 3 |  |
| 47 | Tooth 44 has an addition effect mesially and distally, the root tip cannot be assessed | 2 |  |
| 48 | Tooth 45 has an addition effect mesially and distally, the root tip cannot be assessed | 2 |  |
| 49 | Tooth 46 has an addition effect mesially, the root tip cannot be assessed | 2 |  |
| 50 | Tooth 47 has a whitened distal and a suspect PA fissure enlargement at the tip of the root | 2 |  |
|  | Tooth 48 retained and displaced horizontally in the mesial direction, brightening between the tooth crown and the mesio-caudal bone, the root tip not visible | 3 |  |
| **Additional criteria:** | | | |
|  | Use of technical language | 4 |  |
|  | Consideration of the correct order | 4 |  |
|  | Total: | 100 |  |
